# Supplementary material for: Integration of metabolomics and chemometrics with in-silico and in-vitro approaches to unravel SARS-Cov-2 inhibitors from South African plants
Source: PLoS One. 2025 Mar 26;20(3):e0320415. doi: 10.1371/journal.pone.0320415 (PMC11940557; doi:10.1371/journal.pone.0320415)
Supplement: S1 Fig — (DOCX) [file pone.0320415.s001.docx]

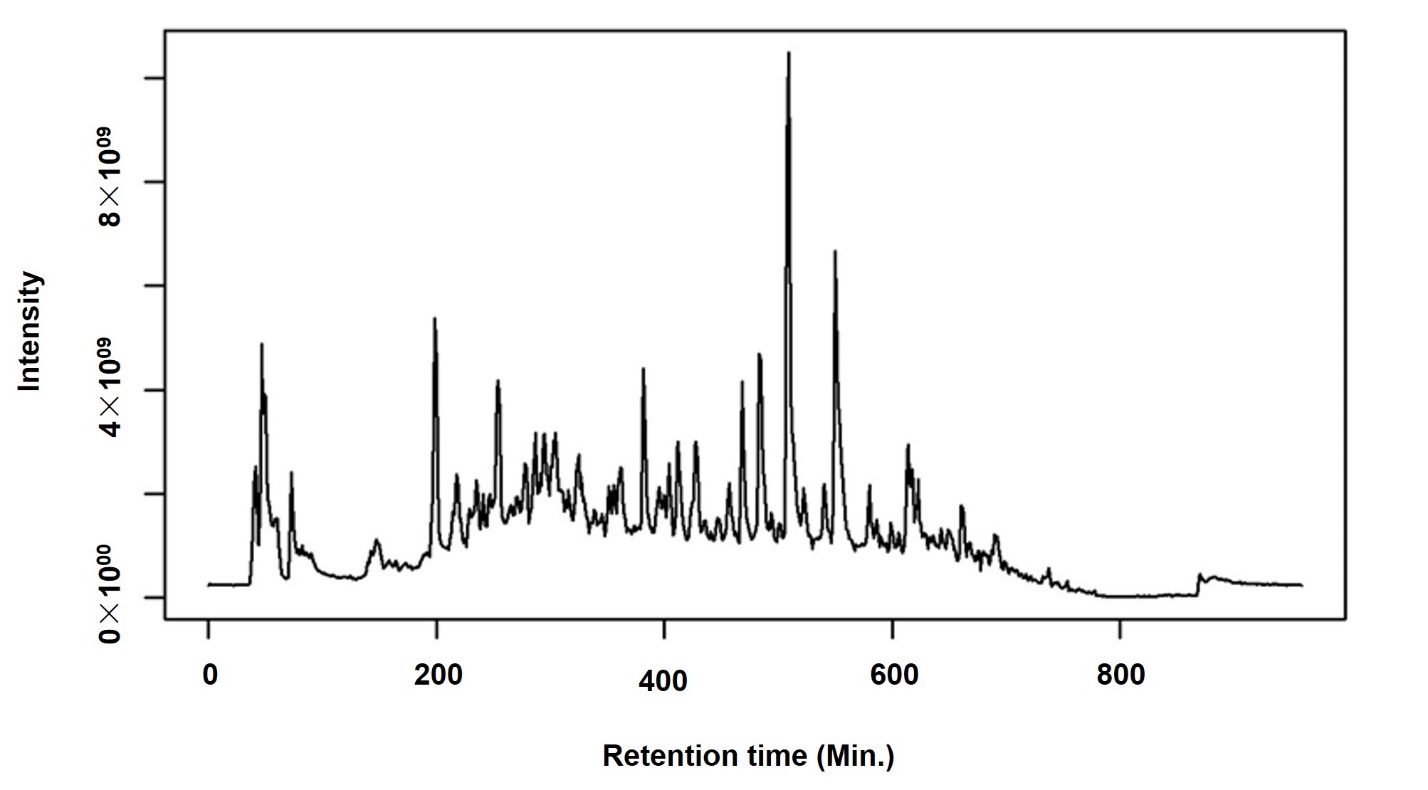


**(a)**


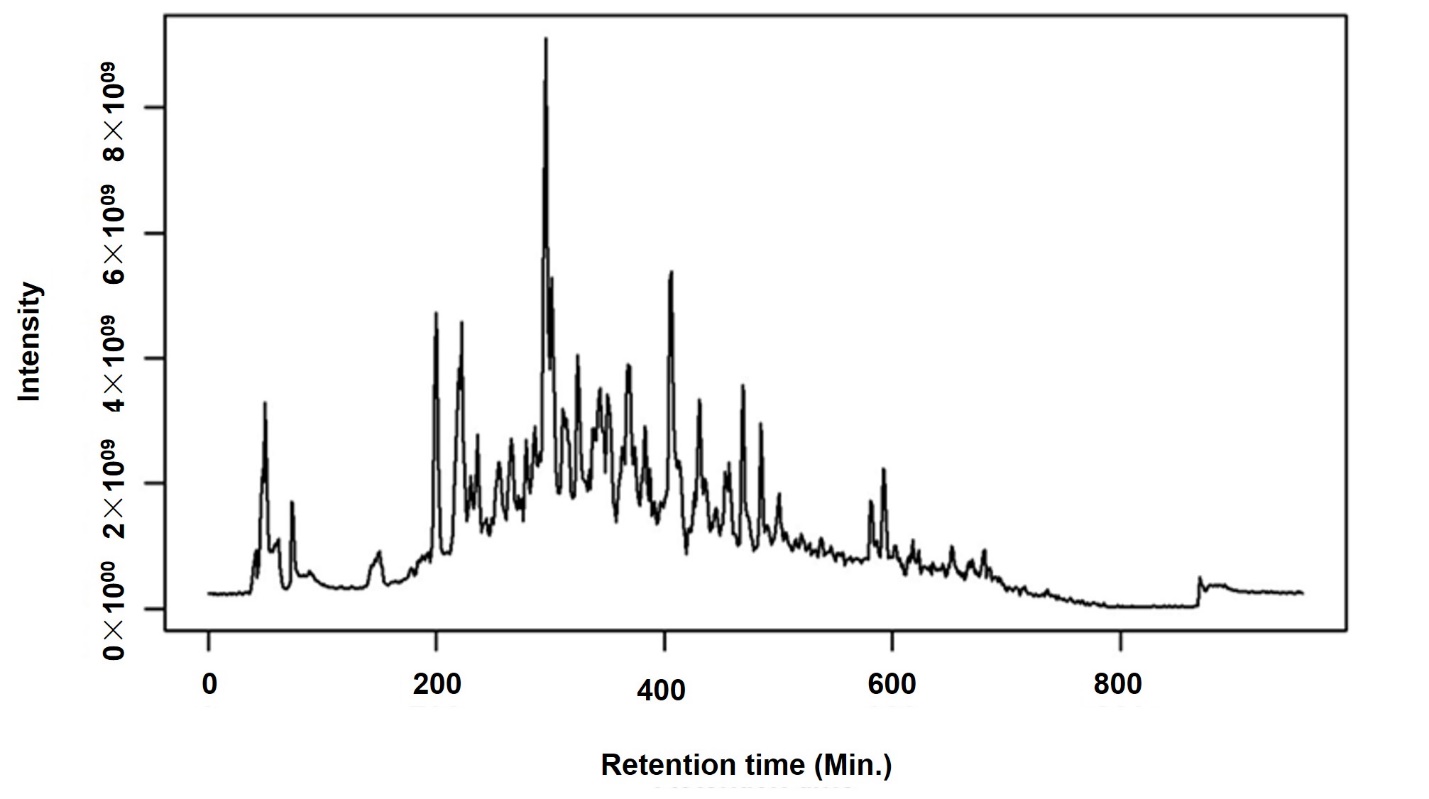


**(b)**

**S1 Fig.** ESI in positive ionization mode depicting base peak intensity chromatograms of (**a**) *A. annua* and (**b**) *A. afra* samples.
