# Supplementary material for: Integration of metabolomics and chemometrics with in-silico and in-vitro approaches to unravel SARS-Cov-2 inhibitors from South African plants
Source: PLoS One. 2025 Mar 26;20(3):e0320415. doi: 10.1371/journal.pone.0320415 (PMC11940557; doi:10.1371/journal.pone.0320415)
Supplement: S1 Table — (DOCX) [file pone.0320415.s002.docx]

**S1 Table.** UPLC-MS/MS distinguished metabolites in the extracts of *A. annua* and *A. afra*.

| **Compound name** | **RT [min]** | **m/z** | **HMDB_ID/** **ChemSpider ID** | **Formula** | **Monoisotopic Mass** | **Delta (ppm)** | **Log^2^**  **(FC)** | **Log^10^**  **(*p* value)** | **VIP** |
| --- | --- | --- | --- | --- | --- | --- | --- | --- | --- |
| 2,4-Pentadienal | 10.135 | 83.04964 | HMDB0031597 | C5H6O | 82.04186 | 6 | 3.20 | 1.74 | 1.02 |
| Fomepizole | 2.75 | 83.06094 | CSID3289 | C4H6N2 | 82.0531 | 7 | 3.50 | 8.80 | 1.52 |
| Piperidine | 1.227 | 86.09691 | HMDB0034301 | C5H11N | 85.08915 | 6 | 4.59 | 5.69 | 1.73 |
| Hydroxypropionic acid | 5.787 | 91.03946 | HMDB0000700 | C3H6O3 | 90.03169 | 5 | 0.78 | 1.53 | 0.33 |
| Methylpyrazine | 2.893 | 95.06079 | HMDB0033112 | C5H6N2 | 94.0531 | 4 | 5.70 | 5.46 | 1.94 |
| 1,3-Hexadien-3-amine | 1.032 | 98.09678 | CSID67029750 | C6H11N | 97.08915 | 4 | 0.03 | 1.00 | 0.04 |
| Valerolactone | 1.024 | 101.06 | HMDB0250981 | C5H8O2 | 100.0524 | 3 | 0.18 | 2.97 | 0.30 |
| Acetoacetic acid | 3.178 | 103.0393 | HMDB0000060 | C4H6O3 | 102.0317 | 3 | 0.43 | 1.32 | 0.46 |
| Serine | 0.974 | 106.0502 | HMDB0000187 | C3H7NO3 | 105.0426 | 3 | -1.32 | 4.74 | 0.92 |
| 5-Methyl-2-furancarboxaldehyde | 1.024 | 111.0442 | HMDB0033002 | C6H6O2 | 110.0368 | 2 | -3.99 | 6.10 | 1.62 |
| Pyrrole-2-carboxylic acid | 1.242 | 112.0395 | HMDB0004230 | C5H5NO2 | 111.032 | 2 | 0.76 | 6.06 | 0.69 |
| Cytosine | 0.974 | 112.0507 | HMDB0000630 | C4H5N3O | 111.0433 | 1 | -0.22 | 1.34 | 0.27 |
| Parasorbic acid | 6.178 | 113.0599 | HMDB0301801 | C6H8O2 | 112.0524 | 2 | 0.58 | 1.78 | 0.39 |
| Indole | 2.487 | 118.0653 | HMDB0000738 | C8H7N | 117.0578 | 2 | 0.23 | 2.33 | 0.31 |
| 1(2H)-Pentalenone | 2.502 | 119.0493 | CSID66739022 | C8H6O | 118.0419 | 2 | -0.32 | 5.25 | 0.44 |
| Phthalide | 2.503 | 157.0282 | HMDB0032469 | C8H6O2 | 134.0368 | 14 | 0.32 | 3.46 | 0.41 |
| 2,6-Dimethylaniline | 3.709 | 122.0965 | HMDB0060677 | C8H11N | 121.0891 | 1 | 0.21 | 1.69 | 0.25 |
| 5-Methylcytosine | 6.097 | 126.0676 | HMDB0002894 | C5H7N3O | 125.0589 | 11 | -1.22 | 9.17 | 0.89 |
| xi-4-Hydroxy-4-methyl-2-cyclohexen-1-one | 3.795 | 127.0754 | HMDB0033629 | C7H10O2 | 126.0681 | 0 | 0.14 | 1.22 | 0.13 |
| gamma-Butyrolactone | 3.78 | 128.0706 | HMDB0000549 | C4H6O2 | 86.03678 | 0 | -0.03 | 0.90 | 0.02 |
| Epigoitrin | 0.853 | 130.0322 | HMDB0251849 | C5H7NOS | 129.0248 | 1 | 0.12 | 1.53 | 0.19 |
| Pyroglutamic acid | 0.987 | 130.0498 | HMDB0000267 | C5H7NO3 | 129.0426 | 1 | -0.16 | 0.97 | 0.35 |
| Indol-2-one | 3.251 | 132.0444 | HMDB0253466 | C8H5NO | 131.0371 | 0 | 3.17 | 8.59 | 1.44 |
| 3-Hydroxyproline | 0.97 | 132.0654 | HMDB0245903 | C5H9NO3 | 131.0582 | 1 | 0.18 | 1.68 | 0.24 |
| 5-Aminolevulinic acid | 0.855 | 154.0497 | HMDB0001149 | C5H9NO3 | 131.0582 | 15 | 0.55 | 2.39 | 0.51 |
| 2-Deoxy-ribono-1,4-lactone | 5.407 | 133.0495 | HMDB0033958 | C5H8O4 | 132.0423 | 0 | -0.21 | 0.98 | 0.08 |
| 5-Hydroxyindole | 4.301 | 134.06 | HMDB0059805 | C8H7NO | 133.0528 | 1 | 1.20 | 2.46 | 0.72 |
| 1,3-Dihydro-(2H)-indol-2-one | 4.479 | 134.06 | HMDB0061918 | C8H7NO | 133.0528 | 0 | 1.20 | 5.23 | 0.87 |
| Homocysteine | 0.845 | 136.0436 | HMDB0000742 | C4H9NO2S | 135.0354 | 7 | -1.18 | 6.15 | 0.86 |
| Trigonelline | 0.81 | 138.0547 | HMDB0000875 | C7H7NO2 | 137.0477 | 2 | 0.93 | 7.58 | 0.77 |
| 1,3,4-Oxadiazepine | 2.644 | 138.066 | CSID67028654 | C4H4N2O | 96.03236 | 1 | 10.04 | 7.11 | 2.56 |
| 5-Ethyl-2-methylpyridine | 2.804 | 139.1228 | HMDB0029729 | C8H11N | 121.0891 | 2 | -3.98 | 2.06 | 1.52 |
| Methyl 2-thiofuroate | 0.897 | 143.0188 | HMDB0037762 | C6H6O2S | 142.0089 | 19 | 4.34 | 7.05 | 1.74 |
| 2-Aminocyclohexanecarboxylic acid | 1.229 | 144.1017 | CSID133327 | C7H13NO2 | 143.0946 | 2 | 0.21 | 0.97 | 0.42 |
| Coumarin | 2.503 | 147.0438 | HMDB0001218 | C9H6O2 | 146.0368 | 2 | -0.43 | 4.62 | 0.52 |
| 1H-Indole-2,3-dione | 1.686 | 148.0391 | HMDB0061933 | C8H5NO2 | 147.032 | 1 | 0.17 | 1.13 | 0.15 |
| Glutamic acid | 0.981 | 148.0602 | HMDB0000148 | C5H9NO4 | 147.0532 | 2 | -0.01 | 0.88 | 0.02 |
| Phthalic anhydride | 11.148 | 149.0231 | HMDB0256501 | C8H4O3 | 148.016 | 1 | -0.34 | 3.26 | 0.44 |
| 3-Amino-2-cyclohexenone | 4.936 | 150.0309 | CSID71227 | C6H9NO | 111.0684 | 6 | -4.06 | 6.01 | 1.63 |
| Guanine | 2.26 | 152.0565 | HMDB0000132 | C5H5N5O | 151.0494 | 1 | -1.00 | 5.97 | 0.79 |
| Xanthine | 1.891 | 153.0405 | HMDB0000292 | C5H4N4O2 | 152.0334 | 1 | 2.63 | 4.20 | 1.30 |
| Parvoline | 3.933 | 153.1384 | CSID63917 | C9H13N | 135.1048 | 2 | 3.96 | 4.87 | 1.65 |
| 3-Amino-2,2-dimethylpropanoic acid | 0.92 | 156.0419 | HMDB0245805 | C5H11NO2 | 117.079 | 1 | 3.63 | 5.11 | 1.54 |
| 4-Phenylpyridine | 3.184 | 156.0806 | HMDB0033123 | C11H9N | 155.0735 | 1 | 1.98 | 6.40 | 1.13 |
| 4-Methyl-2-pentenoic acid | 4.046 | 156.1018 | HMDB0031561 | C6H10O2 | 114.0681 | 1 | 0.81 | 2.34 | 0.65 |
| 5-Isothiocyanatoindane | 3.015 | 157.1334 | CSID642931 | C6H13NO | 115.0997 | 1 | -1.64 | 4.75 | 1.01 |
| N-Acetylproline | 0.99 | 158.081 | HMDB0094701 | C7H11NO3 | 157.0739 | 1 | 0.28 | 3.54 | 0.39 |
| 2,8-Quinolinediol | 3.471 | 162.0548 | HMDB0240311 | C9H7NO2 | 161.0477 | 1 | -0.20 | 1.45 | 0.25 |
| Norcamphoric acid | 3.195 | 200.0915 | CSID207959 | C7H10O4 | 158.0579 | 2 | 2.80 | 8.33 | 1.35 |
| 3 Hydroxycoumarin | 4.771 | 163.0387 | HMDB0002149 | C9H6O3 | 162.0317 | 2 | -0.72 | 1.86 | 0.64 |
| Propylpyrazine | 1.311 | 164.118 | HMDB0041571 | C7H10N2 | 122.0844 | 1 | 8.05 | 5.28 | 2.29 |
| 7-Methylguanine | 2.076 | 166.0722 | HMDB0000897 | C6H7N5O | 165.0651 | 1 | 0.21 | 1.35 | 0.13 |
| Ethylparaben | 1.005 | 167.07 | HMDB0032573 | C9H10O3 | 166.063 | 2 | 0.57 | 2.33 | 0.47 |
| Oxoglutaric acid | 5.003 | 169.0129 | HMDB0000208 | C5H6O5 | 146.0215 | 13 | 3.55 | 3.89 | 1.51 |
| Quinolacetic acid | 2.849 | 169.0494 | HMDB0240257 | C8H8O4 | 168.0423 | 1 | -3.61 | 5.11 | 1.53 |
| 4-Phenyl-3(2H)-pyridazinone | 2.954 | 173.0707 | CSID11248873 | C10H8N2O | 172.0637 | 1 | -4.82 | 5.39 | 1.78 |
| 4-Guanidino-1-butanol | 0.787 | 173.1396 | CSID4476579 | C5H13N3O | 131.1059 | 1 | 4.88 | 3.37 | 1.76 |
| 1-Oxo-1H-2-benzopyran-3-carboxaldehyde | 4.626 | 175.0388 | HMDB0030577 | C10H6O3 | 174.0317 | 1 | -2.96 | 3.16 | 1.35 |
| Nicotyrine | 2.672 | 176.1181 | HMDB0255591 | C10H10N2 | 158.0844 | 1 | 5.18 | 4.86 | 1.86 |
| Ascorbic acid | 1.243 | 177.0391 | HMDB0000044 | C6H8O6 | 176.0321 | 1 | -0.26 | 1.24 | 0.22 |
| Anatabine | 2.75 | 178.1337 | CSID10910 | C10H12N2 | 160.1001 | 1 | 8.36 | 6.08 | 2.34 |
| Xanthopterin | 1.278 | 180.0514 | HMDB0259923 | C6H5N5O2 | 179.0443 | 1 | 0.24 | 1.08 | 0.14 |
| 7-Aminomethyl-7-carbaguanine | 1.725 | 180.0877 | HMDB0011690 | C7H9N5O | 179.0807 | 2 | -0.33 | 1.49 | 0.31 |
| Tyrosine | 1.288 | 182.081 | HMDB0000158 | C9H11NO3 | 181.0739 | 1 | 0.44 | 4.50 | 0.51 |
| 4-(1H-Pyrazol-1-yl)-1-butanol | 2.644 | 182.1287 | CSID34452308 | C7H12N2O | 140.095 | 1 | 7.00 | 4.96 | 2.14 |
| Hydroxyphenyllactic acid | 2.501 | 183.065 | HMDB0000755 | C9H10O4 | 182.0579 | 1 | -0.03 | 0.90 | 0.17 |
| Choline sulfate | 0.988 | 184.0635 | HMDB0250194 | C5H13NO4S | 183.0565 | 1 | -3.69 | 4.92 | 1.55 |
| 5-Fluoromethylornithine | 4.624 | 187.0863 | HMDB0245493 | C6H13FN2O2 | 164.0961 | 5 | 3.25 | 7.48 | 1.46 |
| 1,4-Octadien-1-ylbenzene | 8.255 | 187.1479 | CSID30992614 | C14H18 | 186.1409 | 1 | 3.59 | 8.14 | 1.53 |
| Amino(1H-indol-2-yl)acetic acid | 3.17 | 191.0812 | CSID32887546 | C10H10N2O2 | 190.0742 | 1 | -4.41 | 8.26 | 1.78 |
| 5-Methoxytryptophol | 3.196 | 192.1017 | HMDB0001896 | C11H13NO2 | 191.0946 | 1 | 1.45 | 3.29 | 0.92 |
| 5-Allyl-6-methyl-4(1H)-pyrimidinone | 2.32 | 192.1129 | CSID23948557 | C8H10N2O | 150.0793 | 2 | 3.81 | 5.22 | 1.58 |
| Shinanolone | 4.667 | 193.0823 | HMDB0030580 | C11H12O3 | 192.0786 | 19 | -0.08 | 1.51 | 0.14 |
| Tocainide | 4.23 | 193.1335 | HMDB0015189 | C11H16N2O | 192.1263 | 0 | 1.54 | 2.42 | 0.91 |
| 6-Amino-2,4,5-trimethyl-3-pyridinol | 2.096 | 194.1287 | CSID34236082 | C8H12N2O | 152.095 | 1 | 8.26 | 5.40 | 2.32 |
| 3-Hydroxysuberic acid | 2.866 | 232.1191 | HMDB0000325 | C8H14O5 | 190.0841 | 11 | 0.19 | 2.12 | 0.27 |
| 4,7-Megastigmadien-9-ol | 8.59 | 195.1741 | HMDB0038731 | C13H22O | 194.1671 | 1 | -0.67 | 1.33 | 0.19 |
| 2'-Hydroxynicotine | 2.769 | 196.1442 | HMDB0001329 | C10H14N2O | 178.1106 | 1 | 11.10 | 5.86 | 2.70 |
| Xanthone | 4.405 | 197.0594 | HMDB0259922 | C13H8O2 | 196.0524 | 2 | -2.54 | 1.71 | 1.05 |
| Syringic acid | 3.081 | 199.0599 | HMDB0002085 | C9H10O5 | 198.0528 | 1 | -2.41 | 7.76 | 1.25 |
| 3,6,8-Dodecatrien-1-ol | 10.71 | 203.147 | CSID9151298 | C12H20O | 180.1514 | 12 | 3.60 | 6.99 | 1.60 |
| 3,6-Dodecadien-1-ol | 6.947 | 205.1585 | HMDB0031102 | C12H22O | 182.1671 | 11 | 3.77 | 7.19 | 1.57 |
| 3-Indolehydracrylic acid | 3.336 | 206.0809 | HMDB0059765 | C11H11NO3 | 205.0739 | 1 | 1.79 | 2.43 | 0.96 |
| 4,8-Dimethyl-7-hydroxycoumarin | 3.343 | 208.0965 | CSID4512230 | C11H10O3 | 190.063 | 1 | 0.68 | 2.91 | 0.55 |
| 2',3'-Didehydro-2',3'-dideoxycytidine | 4.434 | 210.0884 | HMDB0245545 | C9H11N3O3 | 209.08 | 5 | 0.02 | 0.89 | 0.05 |
| 2-Methylenecyclododecanone | 5.774 | 212.2006 | CSID465753 | C13H22O | 194.1671 | 1 | 5.85 | 5.07 | 1.96 |
| Vanillactic acid | 1.727 | 213.0755 | HMDB0000913 | C10H12O5 | 212.0685 | 1 | -0.60 | 1.02 | 0.30 |
| 6-(2-Hydroxyethoxy)-6-oxohexanoic acid | 3.717 | 213.0755 | HMDB0061681 | C8H14O5 | 190.0841 | 10 | -0.44 | 1.16 | 0.44 |
| 2',3'-Dideoxyuridine | 4.976 | 213.0906 | HMDB0245547 | C9H12N2O4 | 212.0797 | 17 | -3.49 | 6.90 | 1.54 |
| Succinyl proline | 3.047 | 238.0682 | CSID168469 | C9H13NO5 | 215.0794 | 2 | 0.15 | 2.05 | 0.24 |
| N-Lactoylphenylalanine | 4.391 | 238.1069 | HMDB0062175 | C12H15NO4 | 237.1001 | 2 | -1.99 | 3.44 | 1.11 |
| 1-Phenylcyclohexanol | 3.774 | 218.1537 | CSID14582 | C12H16O | 176.1201 | 1 | 5.05 | 6.59 | 1.82 |
| Hydrocotarnine | 4.956 | 222.1121 | HMDB0033701 | C12H15NO3 | 221.1052 | 2 | 0.31 | 1.05 | 0.00 |
| 4-Pyridoxic acid | 5.862 | 225.0906 | HMDB0000017 | C8H9NO4 | 183.0532 | 16 | -5.38 | 7.24 | 1.97 |
| Vomifoliol | 3.517 | 225.1482 | HMDB0303570 | C13H20O3 | 224.1412 | 2 | 2.86 | 6.86 | 1.37 |
| Undecylenic acid | 4.113 | 226.1798 | HMDB0033724 | C11H20O2 | 184.1463 | 2 | 1.75 | 5.63 | 1.06 |
| 3'-Deoxythymidine | 5.839 | 227.1062 | HMDB0246094 | C10H14N2O4 | 226.0954 | 16 | -5.95 | 6.11 | 2.02 |
| 7-Epi-12-hydroxyjasmonic acid | 3.942 | 227.1273 | HMDB0303749 | C12H18O4 | 226.1205 | 2 | -0.87 | 7.94 | 0.75 |
| erythro-4-Hydroxyarginine | 3.109 | 229.0704 | HMDB0034326 | C6H14N4O3 | 190.1066 | 3 | -1.33 | 2.86 | 0.83 |
| Pyroglutamylvaline | 5.782 | 229.1219 | HMDB0094651 | C10H16N2O4 | 228.111 | 16 | -3.47 | 5.31 | 1.50 |
| mescaline | 2.991 | 229.1543 | HMDB0254474 | C11H17NO3 | 211.1208 | 2 | 1.70 | 8.96 | 1.05 |
| 2-Hydroxy-3-phenylcyclohexanone | 3.169 | 232.1329 | CSID30902651 | C12H14O2 | 190.0994 | 11 | 6.43 | 5.96 | 2.05 |
| 2-(2-Furylmethyl)-1-indanol | 3.905 | 237.0907 | CSID40514807 | C14H14O2 | 214.0994 | 9 | -0.56 | 3.59 | 0.55 |
| Pantothenic acid | 9.416 | 237.1402 | HMDB0000210 | C9H17NO5 | 219.1107 | 18 | 0.31 | 4.77 | 0.43 |
| 12-Hydroxydodecanoic acid | 7.277 | 239.1638 | HMDB0002059 | C12H24O3 | 216.1725 | 9 | 3.20 | 2.50 | 1.38 |
| 3'-Amino-3'-deoxythimidine | 4.038 | 242.117 | HMDB0060750 | C10H15N3O4 | 241.1063 | 14 | -5.09 | 5.64 | 1.85 |
| Thymidine | 5.406 | 243.1011 | HMDB0000273 | C10H14N2O5 | 242.0903 | 14 | -6.83 | 5.66 | 2.13 |
| Phenylalanyl-Glycine | 4.839 | 245.0917 | HMDB0304788 | C11H14N2O3 | 222.1004 | 9 | -1.21 | 2.36 | 0.50 |
| Isopropyl beta-glucoside | 3.5 | 245.1016 | HMDB0032705 | C9H18O6 | 222.1103 | 9 | 0.84 | 2.19 | 0.58 |
| Germacrone-13-al | 4.513 | 250.1796 | HMDB0036881 | C15H20O2 | 232.1463 | 2 | -6.74 | 5.39 | 2.10 |
| Piperdial | 6.936 | 251.1638 | HMDB0035798 | C15H22O3 | 250.1569 | 2 | 1.84 | 3.80 | 1.07 |
| Muramic acid | 3.206 | 252.1087 | HMDB0003254 | C9H17NO7 | 251.1005 | 4 | 0.81 | 1.64 | 0.40 |
| 1-(Ribofuranosyl)indoline | 2.641 | 252.1226 | CSID67029342 | C13H17NO4 | 251.1158 | 2 | 0.00 | 0.86 | 0.16 |
| 2-Hydroxyacorenone | 9.271 | 259.1664 | HMDB0030916 | C15H24O2 | 236.1776 | 2 | 4.60 | 6.34 | 1.74 |
| Keto-3-deoxy-manno-octulosonic acid | 3.079 | 261.0601 | HMDB0244292 | C8H14O8 | 238.0689 | 8 | -0.74 | 3.68 | 0.66 |
| 11-Dodecenoic acid | 4.664 | 262.1797 | HMDB0032248 | C12H22O2 | 198.162 | 7 | 3.26 | 6.66 | 1.46 |
| 9-Pentadecenoic acid | 10.834 | 263.2002 | HMDB0029765 | C15H28O2 | 240.2089 | 8 | 0.73 | 2.59 | 0.60 |
| Risbitin | 5.609 | 264.1954 | HMDB0302980 | C14H22O2 | 222.162 | 2 | 1.51 | 5.28 | 0.97 |
| Perlolyrine | 4.972 | 265.0967 | HMDB0030327 | C16H12N2O2 | 264.0899 | 2 | 1.22 | 5.96 | 0.88 |
| Artemorin | 7.694 | 266.1745 | HMDB0302701 | C15H20O3 | 248.1412 | 2 | 6.29 | 6.02 | 2.03 |
| Linamarin | 1.312 | 270.0944 | HMDB0033699 | C10H17NO6 | 247.1056 | 2 | 1.70 | 4.93 | 1.03 |
| Apigenin | 5.177 | 271.0596 | HMDB0002124 | C15H10O5 | 270.0528 | 2 | -0.07 | 0.90 | 0.15 |
| 1-Hydroxyacorenone | 7.673 | 273.1456 | HMDB0030917 | C15H22O3 | 250.1569 | 0 | 3.19 | 3.70 | 1.43 |
| 5-(Hydroxymethyl)cytidine | 2.821 | 274.1027 | CSID32720391 | C10H15N3O6 | 273.0961 | 2 | 2.00 | 8.88 | 1.14 |
| 3alpha-Hydroxyoreadone | 6.425 | 275.1248 | HMDB0036047 | C14H20O4 | 252.1362 | 2 | 5.16 | 5.71 | 1.84 |
| 2',3'-Dideoxyadenosine | 6.718 | 277.1405 | HMDB0245544 | C10H13N5O2 | 235.1069 | 1 | 4.34 | 10.81 | 1.70 |
| Glutaminylleucine | 3.04 | 277.1906 | HMDB0028801 | C11H21N3O4 | 259.1532 | 13 | -0.31 | 2.97 | 0.41 |
| Glycyl-Tryptophan | 3.196 | 279.1448 | HMDB0028852 | C13H15N3O3 | 261.1113 | 1 | 1.17 | 2.22 | 0.71 |
| 5-Methyldeoxycytidine | 2.036 | 283.1396 | HMDB0002224 | C10H15N3O4 | 241.1063 | 2 | 4.39 | 5.31 | 1.69 |
| 3-Hydroxy-2-oxobutyl nonanoate | 6.094 | 286.2007 | CSID67171193 | C13H24O4 | 244.1675 | 3 | 8.00 | 5.19 | 2.34 |
| Kaempferol | 4.724 | 287.0542 | HMDB0005801 | C15H10O6 | 286.0477 | 3 | -1.17 | 5.40 | 0.87 |
| 4-Hydroxycyclohexylcarboxylic acid | 4.619 | 289.1637 | HMDB0001988 | C7H12O3 | 144.0786 | 3 | 0.51 | 1.29 | 0.29 |
| Prodolic acid | 5.075 | 296.1276 | HMDB0256794 | C16H19NO3 | 273.1365 | 6 | 0.16 | 2.13 | 0.25 |
| Stearidonic acid | 9.676 | 299.1999 | HMDB0006547 | C18H28O2 | 276.2089 | 6 | 2.23 | 2.82 | 1.15 |
| Pollenin A | 4.254 | 303.0493 | HMDB0303704 | C15H10O7 | 302.0427 | 2 | 0.84 | 3.45 | 0.69 |
| 3'-C-Ethynylcytidine | 3.174 | 309.1174 | HMDB0252093 | C11H13N3O5 | 267.0855 | 6 | -0.32 | 3.32 | 0.43 |
| Lactucin | 3.633 | 318.1329 | HMDB0035814 | C15H16O5 | 276.0998 | 2 | -5.38 | 3.37 | 1.88 |
| 2'-Deoxy-5-formylcytidine | 1.99 | 319.0993 | CSID10291642 | C10H13N3O5 | 255.0855 | 8 | -1.64 | 4.84 | 1.03 |
| N2-Galacturonyl-lysine | 6.257 | 323.1482 | HMDB0033105 | C12H22N2O8 | 322.1376 | 10 | -6.46 | 5.55 | 2.06 |
| 7-Methylinosine | 1.031 | 325.139 | HMDB0003950 | C11H15N4O5 | 283.1042 | 2 | -6.80 | 5.12 | 2.11 |
| 9,10-DiHODE | 4.767 | 330.2632 | HMDB0010221 | C18H32O4 | 312.2301 | 2 | 0.00 | 0.86 | 0.01 |
| Aflatoxin G2 | 6.708 | 331.0804 | HMDB0030475 | C17H14O7 | 330.074 | 2 | -7.62 | 5.35 | 2.23 |
| dIMP | 4.25 | 333.0597 | HMDB0006555 | C10H13N4O7P | 332.0522 | 1 | 1.04 | 5.26 | 0.80 |
| Protocatechuic acid 4-glucoside | 2.983 | 339.0679 | HMDB0303826 | C13H16O9 | 316.0794 | 2 | -5.71 | 4.76 | 1.96 |
| 2-Methylguanosine | 4.679 | 339.1407 | HMDB0005862 | C11H15N5O5 | 297.1073 | 1 | -2.39 | 3.89 | 1.23 |
| Glutamyllysine | 2.35 | 339.1656 | HMDB0004207 | C11H21N3O5 | 275.1481 | 5 | 5.59 | 5.14 | 1.91 |
| 6b-Hydroxymethandienone | 10.138 | 339.1922 | HMDB0005832 | C20H28O3 | 316.2038 | 3 | 0.61 | 6.10 | 0.62 |
| 1-O-Caffeoyl-beta-glucose | 5.172 | 343.1016 | HMDB0302440 | C15H18O9 | 342.0951 | 2 | -0.93 | 2.53 | 0.68 |
| 6-Ketoestriol | 6.526 | 344.1848 | HMDB0000530 | C18H22O4 | 302.1518 | 3 | -4.86 | 4.72 | 1.78 |
| Bisoprolol | 5.313 | 348.2162 | HMDB0014750 | C18H31NO4 | 325.2253 | 5 | 3.60 | 1.93 | 1.42 |
| Glucitol-lysine | 5.161 | 352.211 | HMDB0252764 | C12H26N2O7 | 310.174 | 9 | 5.03 | 8.06 | 1.83 |
| MG(18:3/0:0/0:0) | 8.725 | 353.2678 | HMDB0011570 | C21H36O4 | 352.2614 | 2 | 1.18 | 7.12 | 0.87 |
| 6'-Hydroxyenterolactone | 4.893 | 356.1482 | HMDB0041697 | C18H18O5 | 314.1154 | 3 | -1.54 | 3.51 | 0.97 |
| Nicotine glucuronide | 1.84 | 356.1808 | HMDB0001272 | C16H22N2O6 | 338.1478 | 2 | 8.28 | 5.27 | 2.33 |
| Prostaglandin A2 | 7.707 | 357.2028 | HMDB0002752 | C20H30O4 | 334.2144 | 2 | 1.29 | 1.51 | 0.54 |
| 4',5,7-Trihydroxy-6-prenylflavanone | 3.227 | 358.164 | HMDB0037247 | C20H20O5 | 340.1311 | 3 | -9.01 | 5.66 | 2.43 |
| 3-Epinobilin | 3.983 | 364.2109 | HMDB0036690 | C20H26O5 | 346.178 | 3 | -6.27 | 3.87 | 2.03 |
| Zeranol | 4.259 | 364.2109 | HMDB0032702 | C18H26O5 | 322.178 | 3 | -5.67 | 4.08 | 1.92 |
| Eicosanedioic acid | 10.522 | 365.2678 | HMDB0242141 | C20H38O4 | 342.277 | 4 | -1.17 | 6.43 | 0.86 |
| Cibaric acid | 4.971 | 366.2268 | HMDB0038580 | C18H28O5 | 324.1937 | 2 | 4.11 | 6.22 | 1.64 |
| 3-Feruloyl-1,5-quinolactone | 4.631 | 373.091 | HMDB0029289 | C17H18O8 | 350.1002 | 4 | -0.58 | 3.04 | 0.55 |
| 8-Hydroxygeraniol 8-O-glucoside | 4.457 | 374.2165 | HMDB0035025 | C16H28O7 | 332.1835 | 2 | 1.35 | 6.86 | 0.93 |
| Isocolumbin | 3.074 | 376.1747 | HMDB0036837 | C20H22O6 | 358.1416 | 2 | -8.16 | 5.14 | 2.31 |
| Lactol | 3.939 | 378.1901 | HMDB0303945 | C20H24O6 | 360.1573 | 3 | -7.67 | 6.15 | 2.24 |
| Hydroxyisonobilin | 3.359 | 380.2059 | HMDB0034475 | C20H26O6 | 362.1729 | 2 | -5.12 | 4.29 | 1.82 |
| Thromboxane B3 | 4.446 | 386.2529 | HMDB0005099 | C20H32O6 | 368.2199 | 2 | -0.93 | 6.88 | 0.77 |
| 2-trans-O-Feruloylglucaric acid | 3.606 | 387.0913 | HMDB0302546 | C16H18O11 | 386.0849 | 2 | -0.73 | 5.66 | 0.67 |
| Cellulose, microcrystalline | 2.156 | 393.136 | HMDB0032197 | C14H26O11 | 370.1475 | 2 | -0.12 | 1.98 | 0.21 |
| 1,2-Anhydridoniveusin | 1.885 | 394.1852 | HMDB0032105 | C20H24O7 | 376.1522 | 2 | -8.65 | 4.75 | 2.38 |
| MG(5-iso PGF2VI/0:0/0:0) | 6.209 | 423.2344 | HMDB0260485 | C21H36O7 | 400.2461 | 2 | 0.79 | 3.14 | 0.67 |
| Kasugamycin | 1.915 | 402.15 | CSID16736502 | C14H25N3O9 | 379.1591 | 4 | 8.18 | 6.45 | 2.31 |
| N-Acetyllactosamine | 2.923 | 406.1337 | HMDB0001542 | C14H25NO11 | 383.1428 | 4 | 0.16 | 4.21 | 0.30 |
| Eupachloroxin | 3.586 | 446.1566 | CSID4444792 | C20H25ClO8 | 428.1238 | 2 | -7.90 | 4.69 | 2.27 |
| Glucosyloxyanthraquinone | 3.844 | 409.0911 | CSID389045 | C20H18O8 | 386.1002 | 4 | 3.73 | 1.96 | 1.20 |
| MG(i-20:0/0:0/0:0) | 14.504 | 409.3281 | HMDB0072854 | C23H46O4 | 386.3396 | 2 | 5.86 | 3.80 | 1.96 |
| Biocytin | 4.469 | 414.2112 | HMDB0003134 | C16H28N4O4S | 372.1831 | 14 | -6.26 | 2.86 | 1.99 |
| Caryoptosidic acid | 2.971 | 415.1204 | HMDB0034249 | C16H24O11 | 392.1319 | 2 | -0.29 | 5.18 | 0.42 |
| Sergliflozin A | 3.22 | 418.1849 | HMDB0258246 | C20H24O7 | 376.1522 | 3 | -7.79 | 6.17 | 2.26 |
| N-Acetyl-9-aminominocycline, (4R)- | 4.66 | 420.2006 | HMDB0259457 | C20H31NO7 | 397.2101 | 3 | -9.55 | 5.38 | 2.50 |
| Enicoflavine | 3.338 | 423.1752 | CSID4444887 | C10H13NO4 | 211.0845 | 2 | 3.64 | 5.67 | 1.54 |
| 3beta-Hydroxy-5-cholestenal | 9.848 | 423.3249 | HMDB0060131 | C27H44O2 | 400.3341 | 4 | 2.58 | 2.90 | 1.21 |
| Eurycomanol | 1.378 | 428.1906 | HMDB0252130 | C20H26O9 | 410.1577 | 2 | -5.56 | 4.93 | 1.90 |
| Eriodictin | 4.559 | 435.1277 | HMDB0037480 | C21H22O10 | 434.1213 | 2 | -0.01 | 0.91 | 0.02 |
| Aloesol 7-glucoside | 4.058 | 438.175 | HMDB0040565 | C19H24O9 | 396.142 | 2 | 3.43 | 6.60 | 1.51 |
| 7a,12a-Dihydroxy-cholestene-3-one | 10.46 | 439.3199 | HMDB0002197 | C27H44O3 | 416.329 | 4 | 2.38 | 3.05 | 1.20 |
| O-Desmethyltramadol glucuronide | 3.259 | 448.1957 | HMDB0060856 | C21H31NO8 | 425.205 | 3 | 0.40 | 1.67 | 0.34 |
| 25-Hydroxyvitamin D3-26,23-lactone | 11.375 | 451.2833 | HMDB0060126 | C27H40O4 | 428.2927 | 3 | 6.77 | 5.89 | 2.13 |
| Catechin 7-glucoside | 3.177 | 453.1383 | HMDB0037949 | C21H24O11 | 452.1319 | 2 | -0.24 | 2.91 | 0.36 |
| Tyromycic acid | 12.521 | 453.3351 | HMDB0035888 | C30H44O3 | 452.329 | 2 | 1.10 | 2.09 | 0.75 |
| Fluocinolone | 2.966 | 454.2062 | HMDB0252347 | C21H26F2O6 | 412.1697 | 6 | -8.07 | 5.42 | 2.30 |
| Persicogenin 3'-glucoside | 4.747 | 479.1532 | HMDB0041398 | C23H26O11 | 478.1475 | 3 | -0.24 | 2.78 | 0.34 |
| LysoPA(18:2/0:0) | 4.868 | 457.2398 | HMDB0007856 | C21H39O7P | 434.2433 | 16 | -2.07 | 3.89 | 1.13 |
| LysoPA(18:0/0:0) | 10.568 | 461.2676 | HMDB0007854 | C21H43O7P | 438.2746 | 8 | 5.34 | 7.38 | 1.87 |
| Davallialactone | 5.955 | 465.117 | HMDB0250883 | C25H20O9 | 464.1107 | 2 | 4.28 | 4.79 | 1.67 |
| DG(20:4-2OH/0:0/2:0) | 8.79 | 475.2682 | HMDB0297002 | C25H40O7 | 452.2774 | 3 | 0.65 | 1.42 | 0.32 |
| Quercetin 3-O-glucuronide | 4.241 | 479.0812 | HMDB0029212 | C21H18O13 | 478.0747 | 2 | 0.99 | 4.63 | 0.79 |
| Persiconin | 4.285 | 479.1539 | HMDB0037482 | C23H26O11 | 478.1475 | 2 | 0.86 | 5.87 | 0.74 |
| 6''-O-Acetylglycitin | 5.524 | 489.1382 | HMDB0039489 | C24H24O11 | 488.1319 | 2 | 4.53 | 4.94 | 1.73 |
| Limonoate a-ring-lactone | 6.644 | 489.2088 | HMDB0302537 | C26H32O9 | 488.2046 | 6 | -1.97 | 2.08 | 1.02 |
| DG(2:0/PGD2/0:0) | 7.165 | 491.2629 | HMDB0296907 | C25H40O8 | 468.2723 | 3 | 1.00 | 2.47 | 0.69 |
| Quercetin 3-O-(6''-acetyl-glucoside) | 3.718 | 507.1124 | HMDB0029271 | C23H22O13 | 506.106 | 2 | 3.46 | 6.04 | 1.50 |
| DG(6 keto-PGF1alpha/2:0/0:0) | 4.095 | 528.3155 | HMDB0296912 | C25H42O9 | 486.2829 | 2 | 2.72 | 7.98 | 1.34 |
| Caryatin glucoside | 4.025 | 529.1331 | HMDB0037352 | C24H26O12 | 506.1424 | 3 | 4.13 | 5.43 | 1.67 |
| LysoPE(22:2/0:0) | 10.093 | 534.3547 | HMDB0011522 | C27H52NO7P | 533.3481 | 1 | 0.45 | 5.25 | 0.52 |
| Phaseolus epsilon | 3.657 | 544.2378 | HMDB0035039 | C25H34O12 | 526.205 | 2 | -0.95 | 3.96 | 0.73 |
| Gluten exorphin B4 | 6.575 | 547.2161 | HMDB0059794 | C24H27N5O9 | 529.1809 | 3 | -5.58 | 7.68 | 1.93 |
| Quercetin 3-(6''-malonyl-glucoside) | 4.937 | 551.102 | HMDB0037368 | C24H22O15 | 550.0959 | 2 | 6.00 | 5.10 | 2.00 |
| 19-Nor-5-androstenediol | 9.685 | 553.4239 | HMDB0004590 | C18H28O2 | 276.2089 | 2 | 1.48 | 5.62 | 0.96 |
| Sesaminol glucoside | 7.259 | 555.1482 | HMDB0041209 | C26H28O12 | 532.1581 | 2 | -6.32 | 6.24 | 2.03 |
| 7-Dehydrologanin tetraacetate | 3.488 | 557.1828 | CSID391585 | C25H32O14 | 556.1792 | 7 | -0.07 | 1.72 | 0.15 |
| Cucumerin A | 2.79 | 575.1571 | HMDB0301967 | C29H28O11 | 552.1632 | 8 | 0.42 | 4.85 | 0.50 |
| Procyanidin | 3.823 | 579.1484 | HMDB0013690 | C30H26O12 | 578.1424 | 2 | -0.62 | 3.18 | 0.59 |
| Desglucocheirotoxol | 13.088 | 591.2582 | HMDB0033828 | C29H44O10 | 552.2934 | 3 | -1.31 | 3.57 | 0.88 |
| Neolicuroside | 3.869 | 592.201 | HMDB0040728 | C26H30O13 | 550.1686 | 2 | -0.61 | 3.18 | 0.59 |
| Marmesin rutinoside | 2.724 | 596.2325 | HMDB0041413 | C26H34O13 | 554.1999 | 2 | 0.53 | 5.95 | 0.58 |
| Apigenin 7-O-diglucuronide | 4.027 | 623.1222 | HMDB0301685 | C27H26O17 | 622.117 | 3 | 0.48 | 6.01 | 0.55 |
| Cholic acid glucuronide | 12.017 | 623.2843 | HMDB0002577 | C30H48O11 | 584.3197 | 2 | 1.61 | 4.25 | 0.99 |
| Hesperidin methylchalcone | 4.557 | 625.2106 | HMDB0253112 | C29H36O15 | 624.2054 | 3 | 0.06 | 1.48 | 0.13 |
| 2''-O-Acetylrutin | 8.193 | 691.1266 | HMDB0039929 | C29H32O17 | 652.1639 | 1 | 4.94 | 6.66 | 1.80 |
| Linalool (8-hydroxydihydro-) | 4.627 | 692.2884 | HMDB0304700 | C32H42O14 | 650.2575 | 4 | 5.32 | 9.34 | 1.87 |
| Tetramethylquercetin 3-rutinoside | 4.052 | 708.2469 | HMDB0039337 | C31H38O16 | 666.216 | 4 | 0.39 | 4.90 | 0.49 |
| Glucoliquiritin apioside | 3.01 | 730.2684 | HMDB0041149 | C32H40O18 | 712.2215 | 18 | -0.26 | 3.37 | 0.37 |
| Hetastarch | 4.045 | 759.2936 | HMDB0253113 | C29H52O21 | 736.3001 | 6 | 0.41 | 4.64 | 0.49 |
| Leonoside A | 4.557 | 788.2936 | HMDB0040342 | C35H46O19 | 770.2633 | 5 | 0.06 | 1.48 | 0.13 |
| PA(i-16:0/PGE2) | 12.248 | 808.465 | HMDB0267751 | C39H69O11P | 744.4578 | 11 | -5.37 | 4.37 | 1.87 |
