# Supplementary material for: Integration of metabolomics and chemometrics with in-silico and in-vitro approaches to unravel SARS-Cov-2 inhibitors from South African plants
Source: PLoS One. 2025 Mar 26;20(3):e0320415. doi: 10.1371/journal.pone.0320415 (PMC11940557; doi:10.1371/journal.pone.0320415)
Supplement: Attachment — Submitted filename: Review 1.docx [file pone.0320415.s004.docx]

Manuscript entitled " Integration of metabolomics and chemometrics with in-silico 2 and in-vitro approaches to unravel SARS-CoV-2 inhibitors 3 from South African plants" submitted to *PLOS ONE*, has been reviewed. The article aligns well with the provided criteria, demonstrating originality, methodological rigor, and sound ethical practices. Further minor edits could enhance clarity in some sections, but overall, it meets the academic standards. Only few points are required:

1) Line 170: grammatical adjustment, correcting capitalized noun that does not require it ("Protease" to "protease").
2) Adding a statement on ethical approval or clarifying if an ethics waiver was obtained would help meet community standards for ethics and research integrity.
3) While data analysis software is named, the availability of raw or processed data isn’t explicitly stated, creating ambiguity about data transparency. A simple statement on data accessibility (e.g., “Data available upon request” or “Data deposited in [repository]”) would address this.
4) in metabolomics, especially with LC-MS (Liquid Chromatography-Mass Spectrometry), compound identification is often labeled as “tentative” unless confirmed with additional analytical methods like nuclear magnetic resonance (NMR) spectroscopy or comparison to known standards. For example, Line 80: “Metabolomics Approach for Identification and quantification of Bioactive Compounds” it would be more accurate to say, “Metabolomics Approach for Tentative Identification and quantification of Bioactive Compounds”.
5) Since the study focuses on specific active compounds like quercetin derivatives, it might be beneficial to explicitly mention any efforts to cross-validate these findings with other methods or databases. For instance, mentioning if the identified compounds’ spectral data were cross-referenced with reputable databases (e.g., Dictionary of Natural Products (DNP), Metlin, HMDB) could enhance confidence in the tentative identification.
6) Several compounds identified in this study, including fomepizole, methyl 2-thiofuroate, 3-amino-2-cyclohexenone, and 3-amino-2,2-dimethylpropanoic acid, are synthetic and not known to occur naturally. Zeranol, while derived from a natural mycotoxin (zearalenone), is itself a semi-synthetic compound. This raises questions about the study’s methodology for profiling natural products. Clarification on the verification steps taken to ensure the accuracy of compound identification would strengthen the study’s conclusions, particularly in the context of natural product research.
