# Supplementary material for: Integration of metabolomics and chemometrics with in-silico and in-vitro approaches to unravel SARS-Cov-2 inhibitors from South African plants
Source: PLoS One. 2025 Mar 26;20(3):e0320415. doi: 10.1371/journal.pone.0320415 (PMC11940557; doi:10.1371/journal.pone.0320415)
Supplement: Attachment — Submitted filename: Response to Reviewers.docx [file pone.0320415.s006.docx]

**Journal: *PLOS ONE***

**Response to Reviewers’ Form**

**PONE-D-24-46570**

**Manuscript title:** Integration of metabolomics and chemometrics with in-silico and in-vitro approaches to unravel SARS-CoV-2 inhibitors from South African plants

The authors would like to thank the reviewers for their in-depth analysis and valuable comments. The reviewer’s and editors recommended revision and besides them are the rebuttal thereof.

| **REVIEWER 1**  General comment: Manuscript entitled " Integration of metabolomics and chemometrics with in-silico 2 and in-vitro approaches to unravel SARS-CoV-2 inhibitors 3 from South African plants" submitted to PLOS ONE, has been reviewed. The article aligns well with the provided criteria, demonstrating originality, methodological rigor, and sound ethical practices. Further minor edits could enhance clarity in some sections, but overall, it meets the academic standards. Only few points are required: We thank the reviewer for the feedback. | | | |
| --- | --- | --- | --- |
| **#** | **Reviewer’s comments** | **Response** | **Page no.** |
| 1. | Line 170: grammatical adjustment, correcting capitalized noun that does not require it ("Protease" to "protease"). | We appreciate this pertinent comment. We corrected "Protease" to "protease". | Page 7&8  Line 169 & 179 |
| 2. | Adding a statement on ethical approval or clarifying if an ethics waiver was obtained would help meet community standards for ethics and research integrity. | We appreciate this pertinent comment. Ethical approval and clarification were provided. | Page 3  Line 75-77 |
| 3. | While data analysis software is named, the availability of raw or processed data isn’t explicitly stated, creating ambiguity about data transparency. A simple statement on data accessibility (e.g., “Data available upon request” or “Data deposited in [repository]”) would address this. | We thank you for the pertinent comment. Apart from the provided supporting information. A statement on data accessibility was included under the “Data Availability Statement”  Raw data were also uploaded as Supporting Information files | Page 28  Line 510-512 |
| 4. | In metabolomics, especially with LC-MS (Liquid Chromatography-Mass Spectrometry), compound identification is often labeled as “tentative” unless confirmed with additional analytical methods like nuclear magnetic resonance (NMR) spectroscopy or comparison to known standards. For example, Line 80: “Metabolomics Approach for Identification and quantification of Bioactive Compounds” it would be more accurate to say, “Metabolomics Approach for Tentative Identification and quantification of Bioactive Compounds”. | We appreciated and acknowledged the reviewer for clarity. The word “tentative” was added were applicable in the manuscript as requested. | Page 4 Line 98 |
| 5. | Since the study focuses on specific active compounds like quercetin derivatives, it might be beneficial to explicitly mention any efforts to cross-validate these findings with other methods or databases. For instance, mentioning if the identified compounds’ spectral data were cross-referenced with reputable databases (e.g., Dictionary of Natural Products (DNP), Metlin, HMDB) could enhance confidence in the tentative identification. | We appreciated and acknowledged the reviewer for clarity. The compounds’ spectral data were cross validated and thus reputable databases used for cross referencing were mentioned. | Page 8 Line 190-194 |
| 6. | Several compounds identified in this study, including fomepizole, methyl 2-thiofuroate, 3-amino-2-cyclohexenone, and 3-amino-2,2-dimethylpropanoic acid, are synthetic and not known to occur naturally. Zeranol, while derived from a natural mycotoxin (zearalenone), is itself a semi-synthetic compound. This raises questions about the study’s methodology for profiling natural products. Clarification on the verification steps taken to ensure the accuracy of compound identification would strengthen the study’s conclusions, particularly in the context of natural product research. | We thank the reviewer for the comment. Clarity on the verification steps was provided in terms of identification of compounds. Where untargeted metabolomics can reveal unexpected synthetic compounds that might not have been previously known to exist in a plant, providing a starting point for further investigation and potential drug development. | Page 23 Line 397-400 |

| **REVIEWER 2**  The authors of the provided manuscript evaluated the anti-SARS-Cov-2 activity of identified South African plant metabolites through experimental and computational approaches. This manuscript is relevant, valuable in the field of drug discovery, and with potentiality for high citation. Few points are to be addressed prior publication: We thank the reviewer for the feedback. | | | |
| --- | --- | --- | --- |
| **#** | **Reviewer’s comments** | **Response** | **Page No.** |
| 1. | Authors should elaborate more, providing more details on the target topology and pocket description prior to presenting the docking findings. | We agree that a more detailed explanation of the target topology and pocket description will enhance the clarity and rigor of our study. Below, we have expanded on the methodology used to identify binding pockets, with a focus on the role of P2Rank in this process.  P2Rank’s strength lies in its ability to identify binding sites without relying on predefined templates, making it particularly suitable for novel or less-studied protein targets. In the case of SARS-CoV-2 3CLpro, there is currently no standard drug bound to this protein, which makes template-free methods like P2Rank highly appropriate for identifying potential binding sites. Furthermore, P2Rank has been shown to outperform several existing tools, including Fpocket, SiteHound, MetaPocket 2.0, and DeepSite, in terms of binding site identification success rates (10.1186/s13321-018-0285-8, 10.1007/978-3-319-21233-3_4).  Importantly, P2Rank was able to accurately identify the active site binding pockets of SARS-CoV-2 3CLpro, which are located at residues His41 and Cys145. These findings are consistent with previous studies that have identified these residues as critical for ligand binding and catalytic activity (10.3389/fchem.2024.1336001, 10.1080/16583655.2020.1850002, 10.1007/978-3-031-30691-4_7). This further validates the use of P2Rank in our study, as it successfully identified biologically relevant and druggable regions of the protein. | Page 15 Line 261-262  Page 25 Line 433-440 |
| 2 | Authors should provide a relevant rational for adopting the machine learning-ligand binding pocket prediction rather than the pocket depicted for the co-crystallized SARS-Cov-2 inhibitor (3WL; 5,6,7-trihydroxy-2-phenyl-4H-chromen-4-one). | We acknowledge the importance of aligning our methodology with the co-crystallized SARS-CoV-2 inhibitor (3WL; 5,6,7-trihydroxy-2-phenyl-4H-chromen-4-one) and would like to clarify our rationale as follows:  The use of P2Rank, a machine learning-based tool, was not intended to exclude or contradict the binding pocket depicted for the co-crystallized inhibitor (3WL). Instead, it served as a complementary approach to objectively validate and expand the identification of potential binding sites. **Importantly, the ML-driven prediction confirmed the spatial coordinates of the native ligand’s binding pocket (3WL)** while simultaneously identifying additional druggable regions that may not be evident from crystallographic data alone. This dual validation strengthens the reliability of our findings, as the ML algorithm independently prioritized the same active site residues (e.g., His41 and Cys145) that are critical for 3WL binding (10.3389/fchem.2024.1336001; 10.1007/978-3-031-30691-4_7).  We emphasize that **the ML approach does not disregard crystallographic evidence** but enhances its interpretability in two key ways:   1. **Unbiased Identification:** ML-based tools like P2Rank eliminate potential bias by predicting pockets de novo, without relying on pre-existing ligand coordinates. This is particularly valuable for novel targets like SARS-CoV-2 3CLpro, where over-reliance on a single co-crystallized ligand might overlook alternative binding modes or allosteric sites. 2. **Cross-Validation**: The congruence between the ML-predicted pockets and the 3WL-binding site (Fig. 4) demonstrates that computational predictions align with experimental data. For instance, the high probability scores (>0.8) and conservation scores for the ML-identified pockets overlap with the 3WL-binding residues, reinforcing the biological relevance of both methods.   Furthermore, our goal extended beyond validating known binding sites; we aimed to explore underexplored pockets that could inform the design of next-generation inhibitors. For example, the ML approach highlighted solvent-exposed regions and conserved subpockets that may accommodate structurally diverse ligands, as suggested by recent studies (10.1021/acsmedchemlett.4c00146).  We have revised the manuscript to explicitly state this rationale, emphasizing that the ML methodology supports rather than contradicts the crystallographic data. | Page 15 Line 261-262  Page 26 Line 456-462 |
| 3 | Authors should elaborate more on the ligand-target interaction patterns Bonding should be annotated in terms of bond distances. Specially for Hydrogen bonding, this type of compound-protein polar interaction should be presented within hydrogen bond distances as well as bond angles since hydrogen bond depend on both. Authors should mention the Hydrogen bond angles as well as their distances, since the strength of hydrogen bonding is based on both parameters in a way to ensure the adequacy of optimum hydrogen bonding. | We have provided comprehensive annotations of hydrogen bond distances to describe the ligand-target interactions precisely. As presented in the Results section, we have detailed the hydrogen bond distances (e.g., Cys145 at 3.70 Å, His41 at 4.39 Å, Glu166 at 2.94 Å, etc.), ensuring that these interactions are clearly quantified.  Regarding hydrogen bond angles, our focus was primarily on bond distances, as they are the principal determinant in ligand-protein binding stability within our computational docking approach. While we acknowledge that hydrogen bond strength is also influenced by bond angles, our study did not include explicit angle measurements. | Page 16-17  Line no. 273-304 |
| 4 | The author should provide dissection of the free binding energies MM-GBSA or MM-PBSA calculations estimated from the MD simulation trajectories. This would obtain total energy calculations as well as different energy contribution terms (∆G electrostatic, ∆G van der Waal, ∆G polar solvation, ∆G non-polar SASA solvation,…) to identify the dominant energy terms that can guide further ligand optimization and development. Further, the total free binding energies for compound-target complex should be broken down to the target residue energy contribution to highlight which residues impose the great impact on ligand binding for future targeting and lead optimization | We have provided a comprehensive analysis of the free binding energies using MM-PBSA (Molecular Mechanics Poisson-Boltzmann Surface Area) calculations, based on the MD simulation trajectories. The total binding energy (ΔG_binding) is dissected into its key components, including van der Waals interactions (ΔG_VDW), electrostatic interactions (ΔG_EEL), polar solvation (ΔG_Polar), and non-polar SASA solvation (ΔG_Non-polar SASA). Our findings highlight that 2"-O-acetylrutin exhibits the most stable binding, primarily driven by strong van der Waals interactions and moderate electrostatic effects. Additionally, the analysis of solvation energies further underscores the role of solvent effects in stabilizing the ligand-receptor complex. By focusing on MM-PBSA, we have identified the dominant energy terms that influence binding stability, providing valuable insights for future ligand optimization | Page 18-19  Line 325-339 |
| 5 | Authors are advised to provide snap shoot of the MD simulated ligand-protein complex at different time intervals (example 0ns, 20ns, 40ns, and/or 50ns) to track the main conformational/orientation changes for both the compound and surrounding residues over time. | We appreciate the reviewer’s suggestion, Taking snapshots of a ligand-protein complex during an MD simulation at different time intervals is challenging because of the inherent dynamic nature of the system, where molecules are constantly moving and changing conformation, meaning that capturing a representative snapshot at any given moment can be difficult, especially when trying to represent the full range of possible interactions over a simulation trajectory. |  |
| 6 | Based on the study results, what are the take-away messages. Authors are advised to highlight the suggested structural modifications that would improve the compound’s biological activities based on the in silico findings. These insights would be beneficial for guiding further lead optimization and development. | We appreciate the reviewer’s suggestion, and to elaborate on the key take-away messages from our study and to highlight potential structural modifications that could enhance the biological activities of the identified compounds. Below, we have provided a comprehensive discussion addressing these points, which has been incorporated into the revised manuscript.  1. Optimization of the Quercetin Core for Enhanced Binding: Modifications at the C-3, C-7, and C-4' positions with hydroxyl, methoxy, or fluorine groups may strengthen hydrogen bonding interactions, thereby increasing binding stability and selectivity.  2. Introduction of Hydrophobic Substituents for Improved Membrane Permeability: Adding alkyl or aryl groups could increase lipophilicity, leading to better drug absorption and permeability.  3. Glycosidic Stability Enhancement: Since 2"-O-acetylrutin demonstrated the strongest inhibitory activity, modifying the acetyl group to a more hydrolysis-resistant moiety (e.g., ether or carbamate) could improve metabolic stability and extend its biological half-life.  4. Enhancement of π-π Stacking Interactions: The addition of electron-donating groups may enhance π-π stacking interactions within the binding pocket, further improving ligand stability.  5. Solubility Improvement Strategies: As quercetin derivatives generally suffer from poor water solubility, we propose utilizing prodrug approaches such as phosphate ester derivatives or phospholipid-based formulations (e.g., Quercetin Phytosome®), which have been shown to improve bioavailability by up to 20-fold (Francesco et al., 2020; DOI: 10.23736/S2724-5985.20.02771-3). | Page 26-27 line 456-483 |
| 7 | Finally, concerning the conclusion, authors are advised to elaborate more on the future of this work? Will you broaden the scope to another plant source and/or drug target? What are the study limitations and what approaches could be conducted to further address them? | This study opens several avenues for future research, particularly in broadening the scope to additional plant-derived bioactive compounds and alternative viral targets. While our study focused on quercetin derivatives from selected plant sources, future investigations will explore structurally related flavonoids and polyphenolic compounds from other medicinal plants known for their antiviral properties. Promising candidates such as kaempferol derivatives, epigallocatechin gallate (EGCG), and myricetin analogs have shown significant potential against viral proteases and could be screened for their inhibitory effects on SARS-CoV-2 3CLpro, papain-like protease (PLpro), and RNA-dependent RNA polymerase (RdRp). Expanding the study to cover these compounds will help identify additional lead molecules with enhanced antiviral activity, drug-like properties, and bioavailability.  Additionally, while the current study focuses on 3C-like protease (3CLpro) as the primary target, we recognize the importance of broadening the scope to other critical viral targets involved in SARS-CoV-2 replication. Future research will investigate the potential of quercetin derivatives in inhibiting the spike protein (S), helicase (nsp13), and PLpro, which play crucial roles in viral entry and immune evasion. Targeting multiple pathways within the viral life cycle may increase the therapeutic efficacy of these compounds and reduce the likelihood of viral resistance.  Study Limitations and Future Approaches  We acknowledge that while the present study provides strong in silico and in vitro evidence supporting quercetin derivatives as potential SARS-CoV-2 inhibitors, several limitations should be addressed in future work. First, the computational docking and molecular dynamics (MD) simulations, although reliable, are predictive models and require further experimental validation through crystallographic studies (X-ray or cryo-EM) to confirm the exact binding conformations and interactions of the compounds within the active site of 3CLpro. | Page 27  Line: 485-503 |

**Journal Requirements:**

| **Academic editor comments** |  | Page & Line |
| --- | --- | --- |
| 1. Please ensure that your manuscript meets PLOS ONE's style requirements, including those for file naming. The PLOS ONE style templates can be found at  https://journals.plos.org/plosone/s/file?id=wjVg/PLOSOne_formatting_sample_main_body.pdf and  [https://journals.plos.org/plosone/s/file?id=ba62/PLOSOne_formatting_sample_title_authors_affiliations.pdf](https://track.editorialmanager.com/CL0/https:%2F%2Fjournals.plos.org%2Fplosone%2Fs%2Ffile%3Fid=ba62%2FPLOSOne_formatting_sample_title_authors_affiliations.pdf/1/010f0193d27fed22-e58fe240-147c-43f3-816a-d61c55435c72-000000/XSCcrI9unzON5RjUED7YuxWGKKkR1wCMSWsOJOxzAnU=189) | We thank the academic editor for the comment it was addressed. However. We are uncertain about the front style and size of the main texts. | See the manuscript |
| 2. Thank you for stating the following financial disclosure: [South African Medical Research Council, grant number MKOLO24/25]. Please state what role the funders took in the study. If the funders had no role, please state: "The funders had no role in study design, data collection and analysis, decision to publish, or preparation of the manuscript." If this statement is not correct you must amend it as needed. Please include this amended Role of Funder statement in your cover letter; we will change the online submission form on your behalf. | Kindly include the following statement and change the online submission form on my behalf:  "The funders had no role in study design, data collection and analysis, decision to publish, or preparation of the manuscript."  Kindly note the acknowledgement were changed. | Page 30  Line 320-321 |
| 3. Thank you for stating the following in the Acknowledgments Section of your manuscript: [This research was funded by the South African Medical Research Council, grant number MKOLO24/25” and “The APC was funded by South African Medical Research Council”] We note that you have provided funding information that is not currently declared in your Funding Statement. However, funding information should not appear in the Acknowledgments section or other areas of your manuscript. We will only publish funding information present in the Funding Statement section of the online submission form. Please remove any funding-related text from the manuscript and let us know how you would like to update your Funding Statement. Currently, your Funding Statement reads as follows: [South African Medical Research Council, grant number MKOLO24/25] Please include your amended statements within your cover letter; we will change the online submission form on your behalf | The funding information was removed from the acknowledgements section. Thus, kindly include and update the following statement under Funding Statement:  “This research was funded by the South African Medical Research Council, grant number MKOLO24/25” and “The APC was funded by South African Medical Research Council” |  |
| 4. We note that your Data Availability Statement is currently as follows: [All relevant data are within the manuscript and its Supporting Information files] Please confirm at this time whether or not your submission contains all raw data required to replicate the results of your study. Authors must share the “minimal data set” for their submission. PLOS defines the minimal data set to consist of the data required to replicate all study findings reported in the article, as well as related metadata and methods (https://journals.plos.org/plosone/s/data-availability#loc-minimal-data-set-definition). For example, authors should submit the following data: - The values behind the means, standard deviations and other measures reported; - The values used to build graphs; - The points extracted from images for analysis. Authors do not need to submit their entire data set if only a portion of the data was used in the reported study. If your submission does not contain these data, please either upload them as Supporting Information files or deposit them to a stable, public repository and provide us with the relevant URLs, DOIs, or accession numbers. For a list of recommended repositories, please see https://journals.plos.org/plosone/s/recommended-repositories. If there are ethical or legal restrictions on sharing a de-identified data set, please explain them in detail (e.g., data contain potentially sensitive information, data are owned by a third-party organization, etc.) and who has imposed them (e.g., an ethics committee). Please also provide contact information for a data access committee, ethics committee, or other institutional body to which data requests may be sent. If data are owned by a third party, please indicate how others may request data access. | The following supporting information was provided. **S1 Fig.** ESI in positive ionization mode depicting base peak intensity chromatograms of (**A**) *A. annua* and (**B**) *A. afra* samples.  **S1 Table.** UPLC-MS/MS distinguished metabolites in the extracts of *A. annua* and *A. afra*.  **Raw data was further provided (Excell sheet**) | Page 28  Line 510-11 |
| 5. Please remove your figures from within your manuscript file, leaving only the individual TIFF/EPS image files, uploaded separately. These will be automatically included in the reviewers’ PDF**.** | Thank you, figures were removed within the manuscript file and uploaded individually as TIFF files images. | Please see the manuscript |
